# Organizing Safe Transitions from Intensive Care

**DOI:** 10.1155/2014/175314

**Published:** 2014-03-24

**Authors:** Marie Häggström, Britt Bäckström

**Affiliations:** ^1^Department of Health Sciences, Mid Sweden University, 85170 Sundsvall, Sweden; ^2^Mid Sweden University, 85170 Sundsvall, Sweden

## Abstract

*Background*. Organizing and performing patient transfers in the continuum of care is part of the work of nurses and other staff of a multiprofessional healthcare team. An understanding of discharge practices is needed in order to ultimate patients' transfers from high technological intensive care units (ICU) to general wards. *Aim*. To describe, as experienced by intensive care and general ward staff, what strategies could be used when organizing patient's care before, during, and after transfer from intensive care. *Method*. Interviews of 15 participants were conducted, audio-taped, transcribed verbatim, and analyzed using qualitative content analysis. *Results*. The results showed that the categories *secure*, *encourage,* and *collaborate* are strategies used in the three phases of the ICU transitional care process. The main category; a safe, interactive rehabilitation process, illustrated how all strategies were characterized by an intention to create and maintain safety during the process. A three-way interaction was described: between staff and patient/families, between team members and involved units, and between patient/family and environment. *Discussion/Conclusions*. The findings highlight that ICU transitional care implies critical care rehabilitation. Discharge procedures need to be safe and structured and involve collaboration, encouraging support, optimal timing, early mobilization, and a multidiscipline approach.

## 1. Introduction

Critically ill patients are often transferred several times to the healthcare chain of care. A patient's journey may begin with transfer from an ambulance to the emergency room and then sometimes continues to surgery and intensive care followed by a general ward. Intensive care is designed and meant for the sickest patients with potential life threats and vital organs dysfunction; it requires advanced monitoring, technique, diagnosis, and treatments. Organizing and performing patient transfers in the continuum of care is part of the work of nurses and other staff of the multiprofessional healthcare team. Whittaker and Ball (2000) argue that it is important to perform the preparations for a transfer to the general ward accurately and correctly. If this is not done, the patient must be readmitted to the intensive care unit (ICU) and be exposed to further stress [1].

### 1.1. Background

The transfer from intensive care to a general ward can be a challenging process. Patients in critical care often have a complex health situation. Additionally they are often not able to express and communicate their own will. This depending on multiple reasons and common causes could be their present sedation or altered mental status [2]. Discharge planning in general is described as a process which should provide continuity of care for patients [1]. In this study, ICU transitional care is defined as care provided before, during, and after the transfer of an ICU patient to another care unit with the aim of ensuring minimal disruption and optimal care for the patient. This care may be provided by ICU nurses, acute care nurses, physicians, and other healthcare professionals [3]. Hence, in this study, care is defined as a mix of nursing care and medical care.

It is important that patients' transfers from the ICU are done properly and at the right time when there is no longer a need for intensive care [4, 5]. Patients want to feel safe and secure both before and after the transfer [6], and they can easily become dependent on the staff [7]. There are studies that describe how patients perceive their safety during transfer and how they feel about their recovery [8], but it has also been reported how perceived physical illness can affect experiences of displacement. Patients sometimes also struggle with feelings of abandonment, vulnerability, helplessness, and unimportance [9]. Ambivalent feelings about the upcoming transfers are also shown to be common; both positive and negative emotions have been reported [9–11].

The care of critically ill patients is often very expensive [12]. Different factors have impact on patients' recovery from intensive care, premorbid state, social, family, psychological, physical status, and employment [13]. The struggle for hospital bed placement is becoming more and more frequent, and nowadays hospitals often are overcrowded, which also implies that the organization of transfers is especially important for patient safety. Discharge guidelines and policies are seen as important in the management of transfers [14, 15] and are also considered an effective management tool to reduce length-of-stay in the ICU and improve the utilization of ICU resources [16, 17]. However, the results of previous studies indicate that discharge planning often lacks guidelines and tends to be ad hoc and influenced by patient acuity [18, 19]. Priorities in ICU may be necessary to enable admission for the most ill patients, leading to unplanned discharges even during night which are related to higher risks. A study by Goldfrad and Rowan (2000) found that the overall ICU mortality is 2–5 times higher if the patient is discharged at night. In their study, the staff estimated that only 44% of these patients were fully ready for the transfer, compared with over 80% of patients who were transferred during the day [20].

### 1.2. Rationale for the Study

Discharging an ICU patient is a complex process. Patients recently discharged from the ICU may be particularly at risk for adverse events [21] and readmissions to ICU. To complete a patient's transfer from a high technological ICU to a general ward, an understanding of discharge practices is needed [19]. Many studies demonstrate the experiences of the transition but there is no clear description of the process and how it could be organized in order to be safe [5–11].

Based on the experiences described by those involved in the process, it can be argued that it is important to learn more about how to enhance and organize ICU transitional care. Therefore, the aim of this study was to describe, as experienced by intensive care and general ward staff, what strategies could be used when organizing patient's care before, during, and after transfer from intensive care.

### 1.3. Theoretical Perspective

Before, during, and after transfer from the ICU to a general ward, patients experience a transition process. The patients are transferred from the context of high technology to the culture of the general ward [3]. The theory of transitions was used as a perspective in this study [22]. The specific process of transition from the ICU to the general ward has become a topic of interest because difficulties that arise during the process have been increasingly frequent. Transitions can be initiated by such events as acute illness or injury, which also explains why the concept is a nursing concern. The process requires a beginning, middle, and end [23] and how the person feels and perceives the situation is critical as the process continues. Transition could result in a feeling of displacement and lack of control over their lives. The situation and time span vary and may consist of short periods or months and years; an example of transition is hospitalization for an acute injury or illness [24].

## 2. Method

### 2.1. Ethical Considerations

The study has been approved by the Northern Ethical Committee in Sweden (D-number 07-159). The first author informed and asked the nurses about participation in the study in accordance with verbal and written criteria. They were informed about confidentiality and their rights to withdraw their participation without giving reason.

### 2.2. Design

The study had a qualitative descriptive approach. As the aim of the study was to describe and illuminate the transition process between ICU and general ward, qualitative content analyses were considered [25, 26].

### 2.3. Data Collection, Setting, and Participants

The study is performed in a Swedish context. The data were also used in a larger study that aimed to generate theories about main concerns in ICU transitional care. Data were collected between 2008 and 2010 in two hospitals located in Sweden with different sizes. The participants were recruited in three ICUs and five general wards specializing in surgical, medical, or general fields. The ICUs were medical, surgical, or general. One of the hospitals had a step-down unit, and the second had no step-down unit. All interviews were conducted by the first author. There were totally 15 individual interviews: seven ICU nurses from three ICUs in Sweden, one anesthesiologist, one EN (enrolled nurse) from an ICU, and six RNs working in different surgical or medical wards in two hospitals. The participants had different genders and lengths of experience (1–25 years); 3 were males and 12 were women. Their ages varied between 25 and 62 years. The staff in general wards was registered nurses and the nurses who worked in the ICU had a one-year specialist certification in intensive care.

The interviews were performed in a quiet place at the unit in the hospital and lasted between 30 and 50 minutes. The focus in this study was on how to organize patient transfer to the ward for further care and rehabilitation and not on patients transferred for palliative end-of-life care. The initial question was broad and open: “Could you please tell me about how the transfer process for ICU patients is organized at your unit and your feelings about it?” Clarifying questions such as “what do you mean,” “when,” “why,” and “can you tell me more about that” were asked when necessary to encourage interviewees to narrate their experiences.

### 2.4. Data Analysis

The data analyses were inspired by Elo and Kyngäs' (2008) description of content analysis [27]. The interviews were all tape-recorded and later verbatim transcribed. At the beginning, the interview text was read several times to get a sense of the whole. The manifest analyses started with sorting the data in content areas: before, during, and after transfer from the ICU to a general ward. This was followed by an open coding with handwritten notes and headings in the text, using as many headings as possible, close to the text, in order to describe all aspects of the content. The data analysis continued with an interpretative process that included sorting similar codes together. With further abstraction, the codes were divided into subcategories and categories with similar incidents named with content-characteristic words that were all relevant to the aim and the research topic. All categories together created a main category expressing a higher level of abstraction [27].

## 3. Results

The results show that the categories* secure*,* encourage*, and* collaborate* are strategies used in the three phases of the ICU transitional care process before, during, and after transfer from the ICU (Table 1).

The main category,* a safe, interactive rehabilitation process*, illustrates how all strategies were characterized by an intention to create and maintain safety during the process. It also included attempts to help the patient reach the best possible condition in the different phases so that their recovery and the rehabilitation proceeded as planned. One nurse stated:

*The goal for our care in this process is that the patients are going to be better…In all ways, both physical and physiological….*

The main category and the strategies also illustrated a three-way interaction in the process: between staff and patient/families, between team members and involved units, and between patient/family and environment.

The first category,* secure*, included activities that aimed at preserving patient safety during the transfer and preventing adverse events immediately after transfer or later. The second category,* encourage*, included activities focused on strengthening, inspiring, and giving hope and courage to patients and their families. It involved matters of support and participation. The third category,* collaborate*, included activities aimed at intertwining the process: coordination, cooperation, and communication between the ICU and the general wards.

### 3.1. Before Transfer

The first phase in ICU transitional care included* secure*, optimizing vital signs and reducing and adjusting the intensive care,* encourage*, promoting self-ability and customizing information, and* collaborate*, communicating and coordinating with the ward and arranging a pretransfer meeting.

#### 3.1.1. Secure

*Optimize Vital Functions.* Securing the patients' care and preserving patient safety during ICU transitional care were expressed as central. One essential issue was to optimize the patient's vital functions prior to transfer, a key component to minimize risk for adverse and readmissions, which were commonly referred to in the interviews. The ICU staff interacted with technology and other senses to assess and perform clinical judgments. The patient's pain relief and vital signs (especially respiration and hemodynamic) were the focus and a point of decision in how and when the patient could be transferred out of intensive care.

*The most important in the transfer process is that the patients' vital signs are stable enough; we observe this extremely carefully.*

The decision for transfer was made by the physicians—mostly by anesthesiologists in consultation with the responsible physician from the ward. However, the ICU nurses felt that they were involved and interacted with the physicians in the decision by either confirming that the patients were stable enough or postponing the transfer if the patient had respiratory issues, fever, or some other problematic clinical symptoms. If the patient was fragile or weak, it was seen as important to have a couple of extra days in the ICU or, if possible, in an intermediary unit—reassuring that intensive care was not needed anymore. This was expressed not only as one important strategy to prevent readmission but also as something that often could not be done. Timing for transfer was crucial, and the interviews revealed that patients should not remain either too long or too short time in the ICU. The nurses expressed that both compromised patient safety; if they were transferred too early, they were not stable enough, and if they were transferred later than needed, it made them more immobilized and susceptible to infections and other symptoms. 

*Reduce and Adjust.* To secure patient care and to prevent adverse events later in the ICU transitional care process, it was necessary to reduce technology and adjust how medication was administrated. Weaning and timing of extubation was one important part, which was expressed as often time-consuming and difficult for less experienced nurses. The staff stated that patients should have time to recover from the state of full respiratory support, proceed to minor support (noninvasive respiratory support), and finally manage to breathe at least 24 hours without respiratory support. It was also expressed as important to reduce and adjust the care for patient safety, both for the prevention for central line infections and for suiting the upcoming level of care and competence at the ward. Not all ward nurses, for example, managed multilumen central lines and the like. In the interview, it was also apparent that technology should be reduced and only used when needed in the ICU since the environment in the ICU could cause trouble for the individual patient. Instead, the use of technology should follow the need for hemodynamic assessment so that the patients and their families became used to fewer observations and monitoring once the patients were transferred.

Adjustments to care were often done in an interaction between ENs, RNs, and physicians and needed to be documented in the patient record to maintain consistency. It was also considered essential that administration of medications was altered to prepare the patients for the upcoming level of care, for example, shifting to sleeping pills instead of infusions prior to transfer.

*I think that patients that we try to wean that have been here a long time also should be used to not have full monitoring—often, there are enough with a pulse oximetry part of the day.*

#### 3.1.2. Encourage

*Promote Self-Ability.* Encouragement and interaction with patients and their families were expressed as essential. This included supporting the patient's own initiative and promoting self-ability, since the ICU period often had a negative impact on the patient's muscle mass and initiative ability. It was expressed that the patient's self-ability in this phase influenced the following phases in the transfer process. The staff meant that the patient's self-ability should be strengthened prior to transfer as a part of their rehabilitation process and as a step toward managing the altered level of care. Promoting the patient's own ability required a lot of persuasion and sensitive interaction with the patient.

The ICU staff tried to support the patients to manage small things, for example, to use the bed alarm so they would be familiar with it later on to receive help at the ward. The staff described how they tried on a daily basis to encourage patients to sit up at the bedside and in chairs so that they would not be immobilized in bed at all times. It was also considered essential and helpful if the patients got early and frequent physiotherapy at the ICU.

*We help the patient to drink water which includes helping them grip the glass and set it to the mouth…We have to tell them that everything is better than doing nothing; you can do it in your own way.*

*Customize the Information.* Encouragement based on providing repeated and customized information to patients and their families was considered by the staff to be an important tool in organizing ICU transitional care. It was important to adjust and customize the information to the specific patient needs and inform the families about time for the upcoming transfer. It was expressed as essential to interact with the families and inform them several times about the progress, care plans, and goals. The ICU and general ward staff expressed that anxiety could be minimized if patients and their families were encouraged and informed repeatedly that their condition was stabilized and that a transfer would soon occur. Some patients and families needed to hear the information often and asked repeatedly about things related to their medical status and upcoming transfer while others asked nothing. It was expressed as important to give them consistent responses that did not differ, which could include visual information about the environment and routines at the ward—preferably in form of a brochure or something similar. The staff experienced that the patients' and/or families' experience of encouragement and participation in their care depended on how well and how clearly the information was given.

*I think we can do even better, especially to work more with individualized information for the families….*

#### 3.1.3. Collaborate

*Communicate and Coordinate.* The staff described the coordination between the ICU and the department to which the patient would be transferred as a foundation for collaboration, especially when the patient had been cared for in the ICU for a long time. Collaborating involved communicating and coordinating between the care units for transferred patients. The communication involved information about the patient needs and preparing the unit about what they should consider when the patient arrived at the ward. In some cases, it also involved care-planning meetings with families and different physicians. This made a common view for the patients' planning, and it was expressed as a wish for mutuality between the ICU and the ward, allotting responsibility and planning for the patients' transfer. Coordination was described as being better if it was done as early as possible, especially if the patient had a long length-of-stay in the ICU. One unit used a liaison nurse—a person with a specific responsibility for collaboration—which was experienced in facilitating communication and planning for specific needs. The nurses expressed that if the patient had specific needs, they must be planned for and taken care of a strategy to manage care must be prepared. Collaboration could also include planning for what room the patient should be placed in at the ward, considering individual needs and, if possible, what nurse should be responsible. One ICU nurse said:

* It is important to communicate early and tell them (the staff at the ward) about every need that the patient may have and not cover or exclude anything. Especially if the patient has been a long time in ICU…It is better to overstate rather than to reduce….*

*Arrange a Pretransfer Meeting.* Collaborating and arranging a pretransfer meeting between ward staff and the patients and their families before transfer was expressed as one appreciated strategy. This was described as extra important when the patient or the family was anxious or if the patient had been cared for in the ICU for a long time and had extended needs. The ward nurses meant that this pretransfer meeting was valuable for them and for the patient, since it was an opportunity to create a contact with the patient and family before transfer. The meeting also provided a possibility to ask questions and get a picture about of the patient's care needs. The meeting was described in two ways: at the ICU or in the general ward, depending on the patient's status. The nurses expressed that when a pretransfer meeting was organized, this helped to intertwine the healthcare chain.

*Right now, for example, we have a gentleman at our ward that was 4-5 months in ICU; he has probably a length-of-stay of 8 months in the hospital! And before he came up, we were down there and introduced us, told him that we would be responsible at the ward! *

### 3.2. The Day of Transfer

This phase was the shortest and only included the actual day of transfer. The strategies in this phase included* secure*, assess and summarize, hand over responsibility, and arrange a safe transfer,* encourage*, give confidence, and* collaborate*, negotiate and facilitate.

#### 3.2.1. Secure

*Assess and Summarize.* The staff expressed that it was important prior to transfer to take time to assess and summarize the patient's discharge status and the different nursing phenomena and actions that had been or should be taken for the patient. This was easier if there was a responsible nurse for the specific patient. If the transfer was not planned in advance, this phase also included examining all the equipment that the patient still had and considering removing measurement and technology specific to intensive care and that were not wanted or needed at the general ward.

*I think it's easier when we have someone who is responsible and keeps up the documentation and prepares for discharge…//Then it will be better and continued updated. We really want the patient's record to be clear and that it is obvious what is planned….*

*Hand over Responsibility.* The actual handover was a time for passing responsibility for the patient. The report was mentioned by the staff to be an essential tool to maintain continuity of care and was experienced as the way to hand over the patient without any loose ends. The staff from the general ward tried to prepare themselves by taking part of patient documents before they got to the ICU, but they did not always have this opportunity.

*I try to keep up reading about the patient before the transfer so I don't have to start from zero knowledge. For example, what surgery they've done, drains, and also quickly check the latest values… So I know what to expect.*

The handover often included a nurse-to-nurse report intended to focus briefly on the history and more specifically on the actual status and the planned care. The handover was experienced in different ways. The ICU nurses expressed that their handover should be adjusted with adequate information but they wanted to tell as much as possible so that no loose ends would be left. Some ward nurses expressed that they wanted less history and more of the actual status with planned X-rays, medications, and IV-fluids as well as more of a description of the patient's own ability and problems. It was also important to know the family situation. Sometimes, the nurses from the general ward felt insecure but did not dare to ask the ICU nurses about the patient, since they did not want to seem incompetent.

*Arrange a Safe Transfer.* Most ICU patients are fragile, and a safe transfer that maintains patient safety was expressed as essential. The patient often received oxygen during the transfer to avoid desaturation, and, sometimes, portable monitors were used to control vital signs. In the interviews, one nurse described an example of adverse events related to a transfer:

*Well, we don't want something critical to happen during the transfer…But I will always remember one time I got to get a patient from ICU… I directly saw that she was hugely fragile and wondered if we really should move her from ICU. But both our physician and ICUs physician insisted, and we took the patient and went away… Immediately when we came out from the elevator, the patient's vital signs were deteriorating. We hurried as soon we could into the ward to get help and had to alarm for assistance…. *

#### 3.2.2. Encourage

*Give Confidence.* The staff experienced that it was essential to give confidence prior to and during transfer and to talk in a positive way with patients and their families. The nurses expressed that some patients were anxious and showed feelings of disconnectedness when leaving the environment where they felt safe and staff they knew. The staff felt that the patients' families also needed to feel that the transfer was a step forward and that it was a positive sign.

*When the day of the transfer comes, it may not be too ridden with anxiety for the patient either - but you have to play down the whole and try to make it easier…It might be a grief to leave the place where you (have) been so long and become better. But you have to try instilling that there is something positive! That you try to focus on that this is an important positive sign that the patient has to be transferred at a ward, emphasize that now is the worst of the crisis over!*

#### 3.2.3. Collaborate

*Negotiate and Facilitate.* Collaboration was described by the staff as essential on the day of transfer and included negotiating a suitable time for transfer and facilitating the transfer for all involved staff members. It was important to seek resolutions for problems and to see each other as equal. The staff expressed that barriers could occur for collaboration that instead complicate the transfer, for example, blaming each other and not trying to understand each other's work situation. Hence, interprofessional respect between the staff at the units was essential and facilitated the process.

The actual day of the transfer was expressed as more or less planned, depending on the acute situation at the unit. The nurses on general wards said that it was obvious that the ICU, with rapid decision-making, led to rapid transfers, sometimes unplanned. They wished to be involved in deciding the time of transfer if possible, a time that suited staff from both the general ward and the ICU. The staff at the general ward considered it extremely important to have the opportunity to prepare when they were about to get a patient from the ICU, since it was often time-consuming. If they knew in time, they could plan their work, which made the process feel easier and safer. When the staff communicated about a suitable time for transfer, they also were informed about specific equipment needed, such as oxygen.

*Most of the times, we cannot influence the time for transfer, but we think that it more often should be possible to communicate about it, make it suit us all….*

### 3.3. After Transfer

The third phase included the strategies* secure*, take charge,* encourage*, create a good encounter and instill hope and courage, and* collaborate*, prepare discharge and follow up.

#### 3.3.1. Secure

*Take Charge.* When the patient arrived at the general ward, the staff stated that it was important to get a grip of the whole situation and to take charge of the patient. Taking charge of the patient included actions important for prevention and maintaining patient safety. It involved an overall view from clinical judgments to take control over the paperwork and plan future care—actions that were described as time-consuming. Depending on the patient's status, the ward nurses spent more or less time on continuing care, for example, supplying oxygen; controlling wounds, drains, and drain holes; monitoring feeding tubes and intravenous lines; calculating fluid balance; and checking vital signs. The nurses expressed that it also was vital that the physician at the ward checked out the ordinations and wrote an updated status in the patient's record so that they had a tool for managing their care.

*It's not just (that) the patient must be in good condition when he or she is discharged from the ICU; the care must also continue with high quality at the general ward.*

Sometimes, it was necessary to change equipment and time for medication to suit the care at the ward. Some of the ward nurses expressed that they felt that it was more often patients with a shorter length-of-stay at the ICU who were hemodynamically unstable and readmitted. According to their experience, patients with longer length-of-stay more often were better optimized in their vital functions. The ward nurses mentioned that their control of vital signs sometimes required them to alarm a specific outreach team. This team included staff from the ICU (nurse/physician) who could initiate treatment for the patient at the ward or decide to readmit the patient to the ICU again. They expressed that the outreach team was a helpful tool and made them feel safer in their care but also meant that it was important to have personal clinical judgment.

*There are always some warning clocks when you are checking vital signs and something is wrong. Sometimes, you can feel that something is breaking out, something will be wrong with the patient… I think it depends of a combination of (your) own intuition and the patient's history….*

#### 3.3.2. Encourage

*Instill Hope and Courage.* Creating a good encounter with the patients and their families at the first meeting (either in the ICU or when arriving at the general ward) was expressed as something that contributed to courage and hope. A good encounter included a personal meeting, introducing the responsible staff, and interacting and supporting individual needs. The ward nurses wanted to have time to communicate and sense needs at the first encounter so that they could calm the patients and their families. They expressed that families often displayed anxiety and had questions about the differences in the environments. It was also important to inform the patient and other family members that staff members were available at the ward even though they were not physically present at the patient's room all the time. It took some time to establish hope and courage, and some were more difficult than others.

*The patients and their families can be in a shock reaction when they arrive, and they still try to process and understand what happened…According to my experience, this is more often seen if the intensive period were short.*

The ward nurses expressed that early mobilization in the ICU was vital for how the patient's rehabilitation process proceeded. The nurses expressed that the patient's own ability to take initiative needed to be supported. Depending on diagnosis, this ability differed, but the ward nurses expressed that the care in the ICU often made patients immobilized and used to others taking care of their hygiene. Nurses in the ward felt that the patients had become apathetic and then stepped aside, as others still were responsible for their body.

*Some can stand on their legs immediately, but some must first be mobilized in the bed—but you can really tell that they've been working with early mobilization in ICU….*

#### 3.3.3. Collaborate

*Prepare Discharge.* Collaboration in this phase included planning for further discharge for the patient, which depended on the individual patient's status and home situation. Sometimes, patients needed extensive planning and interaction with community nurses while other patients could be directly transferred at home without any extended help. The nurses explained that this planning often took time and was an important part of the patient's healthcare chain.

*Follow Up.* The participants expressed that following up the patients after intensive care was important. The ICU and general ward staff often continued their collaboration around the patient following transfer—a strategy that intertwined the ICU transitional care process. Sometimes, a ward visit was made by the ICU staff primarily not only to meet the patient again but also to give the staff an opportunity to ask questions and to give medical advice if needed. In one of the hospitals, patients were further followed up within postintensive care meetings after they had been discharged and left the hospital.

*We try to go up and visit the patient at the ward a few days after the transfer, if we have time; it's appreciated by both the patient and the staff since they may ask if there is something missed.*

## 4. Discussion

### 4.1. Discussion of the Results

The main category of this study showed that strategies used by the staff before, during, and after transfer aimed to contribute to* a safe, interactive rehabilitation process*. The results showed how the strategies—*secure*,* encourage*, and* collaborate*—could be used to enhance and organize ICU transitional care.

Patient safety and interaction with patient, family, environment, and other team members are essential for ICU transitional care. Collaboration intertwines the healthcare chain and is a foundation for ICU transitional care. The main category also illustrates that there is a three-way interaction in the process: between staff and patients/families, between different team members and involved units, and between patient/family and environment. The interaction between environment and patients has also been illuminated by theorists such as Rogers, King, and Nightingale, all of whom claimed that the patient is in constant interaction with his or her environment [28]. The ICU staff interacted with technology as a tool to determine if patients were stable enough for weaning and, further on, for transfer. The staff felt that the environmental differences between the ICU and general wards are often a cause of concern for patients and their families. According to Meleis et al.'s theory of transition, people react differently in transition and processes of change [24]. Therefore, we assumed that the care must be individualized even if there are routines and procedures to follow. Transition stands for a change in health status, role relationships, expectations, or abilities. The transition event is dependent on, for instance, suddenness, personal meaning, and level of well-being [28]. Hence, it can be argued as logical to strive for a strengthening process that considers these needs and involves an active plan for a smoother transition. It is important to clarify and explain to the patient and family that observations and monitoring are reduced and adapted to the patient's current health status, which may reduce the perceived stress during the transitional phase. Overall, the results indicate that the transition experience is dependent on preparation of both patient and families for the first contact with the team at the ward and the time of transfer; therefore, the planning must be taken seriously. Collaboration and planning are essential to take time, especially in complex cases.

#### 4.1.1. Secure

The results showed that the nurses strived for optimal timing for transfer. Timing seemed to be a critical point, and this is confirmed in a study by Garland and Connors (2013). Their study indicated that 30-day mortality increased not only if the patients had to leave earlier than planned from the ICU but also if the transfer was delayed so they had to leave later than was optimal [29]. Our study shows that timing and competence concerning extubation and weaning procedures are essential. The nurses felt that weaning required time and effort to manage. This is in accordance with other researches and confirms that weaning is vital for patient safety. A study by Metnitz et al. (2003) showed that readmission was more often seen when the time between extubation and discharge from the ICU was short [30]. A recent study including patients with traumatic brain injuries showed that reintubations within 48 hours (i.e., extubation failure) were significantly associated with lengthened hospital stays, increased frequency of tracheotomy and of pulmonary complications, worsened functional outcomes, and increased mortality [31]. Intermediary units can be used as a way to reduce care and to prepare patients for the altered level [32, 33], but not all hospitals have this kind of unit. However, ICUs can identify beds at the unit aimed at intermediary care where patient care can proceed but with less technology and staff.

Our study indicates that handover is another crucial link for safe transfer. The necessity of avoiding miscommunication during clinical handovers is also described in several other studies. Miscommunication can lead to risk of prolonged stay, lack of continuity of care, suboptimal patient flow, readmissions, and patient dissatisfaction reports [34, 35]. In a systematic review of Foster and Manser (2012), handovers and transfer of patients in acute care were studied. The results showed that the handover process is multifacetted and can therefore be difficult to compare and evaluate, but they also show that standard report pages are one way to facilitate reporting [36]. Boutilier (2007) concluded that the most important thing in the ICU transitional care process was to systematically communicate the necessary information to the receiving device so that patient safety was not threatened and to ensure the necessary continuity of care [37].

#### 4.1.2. Encourage

The second strategy found in our study,* encourage*, is often mentioned in nursing. To encourage meant to instill hope, identity, and confidence for the patients and their families. The results of this study illustrate the importance of pep talk and supportive strategies to encourage the patient to manage the transition and recover from critical illness. According to the nurses, patients need to feel safe, a result also described by Hupcey (2000) as a psychosocial need for ICU patients [38]. One characteristic of transition is disconnectedness associated with disruption of the links on which the person's feelings of security depend [22]. The nurses in this study wanted to be able to offer customized information prior to transfer since they thought that anxiety could be reduced with information. This can be compared with the result from Strahan and Brown (2004), who found that patients often are anxious over how the transfer process will be done and wish to receive detailed information on what will happen [10]. The nurses expressed that the patients and their families should be a part of the transfer. Presence from relatives can affect the patient's sense of participation and contribute to feelings of being cared for and of safety [39].

It is important to minimize experiences of a gap between the ICU and general wards [14]. Health and illness transitions include the sudden role change that can result from moving from a well state to an acute illness. The results of this study indicate that the patient's role changes when arriving at the general ward—from being more or less passive to being seen as active. Furthermore, patients have a desire for normality and independence and wish to be able to have personal contacts—also seen in a study by Mckinney and Deeny (2002) [8]. The results also illustrate the importance of the team at the receiving ward having the opportunity to devote time to the patient and family when they first arrive. The ward nurses in our study expressed that patients showed mixed feelings about the transfer to the general ward and that they needed to instill hope and courage. Our study indicated that both an immediate and later follow-up after leaving the ICU could be useful, helping patients identify problems and find routes for rehabilitation and support. A study by Schandl (2011) actually showed that multidisciplinary ICU follow-up and the first six months after discharge are most important for follow-up [40].

#### 4.1.3. Collaborate

The results in this study showed that ICU transitional care is a complex, multidisciplinary process that involves collaboration both within the ICU and within other units involved.

The findings of our study show that collaboration includes respectful, functional communication between units and different actions aimed to intertwine the healthcare chain. Effective teamwork and coordination among staff can improve the ICU patient discharge process and also reduce the gap separating ICU and the general ward [14, 21]. Kerfoot et al. [41] claim that multidisciplinary collaboration is necessary to affect patient safety. Our study indicates that team members in their own unit and in other units need to treat each other with respect so everyone ask questions without fear or ridicule contempt. A recent study [42] highlights the importance of communication in acute care settings. The results of their study showed, as did this study, that respect and equality are important.

Previous studies have focused on how a specific liaison nurse can be used to facilitate collaboration. A liaison nurse coordinates the transfer and is helpful for the patients and their families [43, 44].The results of this study indicate that there are benefits to a function like that.

#### 4.1.4. ICU Transitional Care, Recovery, and Critical Care Rehabilitation

The results illuminate that ICU transitional care comprises critical care rehabilitation. Many of the strategies during the process focus not only on identifying and minimizing risks for complications, such as pneumonia and central line infections, but also on strengthening mind and body. ICU transitional care can be compared with other recovery processes. One process with similar characteristics is the recovery process for mental illness. This process is described as an active, unique, nonlinear process with stages and phases. The results of this study follow a model called CHIME: connectedness, hope and optimism, identity, meaning in life, and empowerment [45].

One important part of ICU transitional care is to promote patients' self-care capability and encourage patients' autonomy [46]. This is confirmed by Chick and Meleis (1986), who also claim that transitions are linked to shifts in self-care capability. Orem (1980) writes that independence was recognized by early nursing theorists as important for patients well-being and closely connected to their ability to perform daily activities and meet their own care needs [47]. However, the nature of intensive care and its environment makes independence and autonomy difficult. Hughes (2004) emphasized that the degree of autonomy is connected not only to the ability to actually be independent but also to the healthcare staff's perception and understanding of the need for this assessment [48]. Early mobilization is one strategy to uphold patients' self-care capability [49]. The nurses in this study expressed that early mobilization was felt important not least for the patients' ability to do things for themselves and manage the upcoming care. This can be compared with the results of an intervention study by Korupolu et al. (2011) [50]. They saw that early mobilization in the ICU and a strategy for whole-body rehabilitation in the early stage of critical illness showed better functional outcomes at hospital discharge, a shorter duration of delirium, and more ventilator-free days compared with standard care. McFetridge (2011) discussed the importance of a structured, patient-centered rehabilitation program that patients can follow on their journey from critical care to ward and, finally, through discharge from hospital. McFetridge also claims that interaction in care is essential so as to prevent actions taken from becoming fragmented [51]. Hence, the process should be seamless and transparent for all persons that are involved in the patient's care journey and include a multidiscipline approach and a family perspective.

This study illustrates how the strategies* secure*,* encourage*, and* collaborate* summarize specific actions that can be used as a basis for a patient-centered guideline for ICU transitional care. However, the fact that guidelines on their own are not a solution to minimize the gap between ICU and general wards must be discussed. A cultural gap has been identified between ICU and general wards [14], and a study by van Sluisveld et al. (2013) implies that there are social and cultural barriers to the implementation of guidelines and effective ICU discharge [52]. To summarize, no guidelines in clinical practice are useful if there is low adherence. Also important are resources (time, staffing) and knowledge—essential components in order to manage the organization of a safe transfer process from the ICU to a general ward. Nurses' intention to support patients in ICU transitional care often is balanced against the organization's demands, work stress, and time restraint [53]. A recent study showed that nursing care hours per patient in the ICU and skill that mix significantly contribute to prevention of bloodstream infections and a shorter length-of-stay in the ICU [54]. Duffield et al. (2011) found that caring for an increasing number of patients with complex problems caused stress for nursing staff already facing work overload in wards [55].

### 4.2. Methodological Considerations

Qualitative content analysis was used in this study and was well suited since the aim was to describe experiences of intensive care and general ward staff. The results were derived from data from three ICUs and six general wards and, therefore, other hospitals may have other strategies for ICU transitional care that have not been described in this study. However, the result is in concordance with other researches on managing the process. The first and second author have their own experiences as RNs from both ICU and general wards, and their preconceptions have been bridled by thorough data analysis and discussion, by being open as possible to new perspective. This study involved staff with different professions from different hospitals, in order to enhance the variety of experiences. Both authors were familiar with chosen method. During the analysis they were both part of the process and discussed how to label the codes, subcategories, and categories until agreement. By presenting the process of the analysis and presenting the result with quotes from the text, so it is possible for the readers to create their own value, important for the study's credibility.

## 5. Conclusions

The results of this study conclude that* secure*,* encourage*, and* collaborate*, called the SEC-model, illustrates essential strategies suggested to use when organizing the care before, during, and after transfer from the ICU to a general ward. The result is in concordance with other researches on meeting the needs of patients and families. A recent review described patients' and families' experiences about transfers from ICU; the themes were physical responses, psychological responses, information and communication, safety and security, and the needs of relatives [56]—all of which have been addressed in the SEC-model developed through this study. The significance of this study is also strengthened by a study by Lin et al. (2009), who claim that clarification of guidelines and standardization of discharge decision-making and handover are needed [57]. Research also indicates that the care of acute ill ward patients is suboptimal which implies that this crucial link needs to be safer. According to Massey et al. (2008), suboptimal care at the wards depends on failure to seek advice, failure to appreciate clinical urgency, lack of knowledge, failure of the organization, and lack of supervision [58]. To ensure safe and effective care transitions, strategies are needed to improve shared situational awareness, teamwork, patient flow, and resource efficiency in ICU transitional care [59, 60]. The result indicates that a successful ICU transitional process aims to create an interactive, safe, forward-thinking process influenced by actions that preserve patient safety and promote individualized care. To sum it up, a safe transition involves coordination, optimal timing, early mobilization, participation, and a multidiscipline approach. Also relatives perceive safety and a continuing care as very important in the ICU transitional care process and want to participate [61]. A recommendation for future studies is to explore safe transitions out of the family perspective in a systemic way.

## Figures and Tables

**Table 1:** *Secure*, *encourage*, and *collaborate* strategies in ICU transitional care.

	Generic category/subcategory		
	Secure	Encourage	Collaborate
Before transfer	(i) Optimize vital functions (ii) Reduce and adjust	(i) Promote self-ability (ii) Customize the information	(i) Communicate and coordinate (ii) Arrange a pretransfer meeting

The day of transfer	(i) Assess and summarize (ii) Hand over responsibility (iii) Arrange a safe transfer	Give confidence	Negotiate and facilitate

After transfer	Take charge	Instill hope and courage	(i) Prepare discharge (ii) Follow up

A safe, interactive rehabilitation process.
